# Genetic diversity of dengue virus circulating in the Philippines (2014–2019) and comparison with dengue vaccine strains

**DOI:** 10.1371/journal.pntd.0012697

**Published:** 2024-12-19

**Authors:** John Mark Velasco, Chonticha Klungthong, Piyawan Chinnawirotpisan, Paula Corazon Diones, Maria Theresa Valderama, Susie Leonardia, Wudtichai Manasatienkij, Khajohn Joonlasak, Prinyada Rodpradit, Jennifer Mateo, Vicente Vila, Fatima Claire Navarro, Anthony Jones, Aaron Farmer, Stefan Fernandez

**Affiliations:** 1 Department of Virology, WRAIR-AFRIMS, Bangkok, Thailand; 2 University of the Philippines Manila, Ermita, Manila, Philippines; 3 V Luna General Hospital, Armed Forces of the Philippines Medical Center, Quezon City, Philippines; 4 Office of the Surgeon General, Camp General Emilio Aguinaldo, Quezon City, Philippines; University of Pittsburgh, UNITED STATES OF AMERICA

## Abstract

Dengue virus has four distinct serotypes and the genetic diversity within each of the four serotypes contribute to its complexity. An important aspect of dengue molecular evolutionary studies has been the dissection of the extent and structure of genetic variation among major genotypes within each serotype. It is important to understand the role of dengue genetic variability and its potential role and impact in the effectiveness of the dengue vaccine. Demographic data and blood were collected from patients seen at a tertiary hospital in the Philippines and clinically diagnosed with dengue from 2014–2019. Dengue virus (DENV) RT-PCR was used to confirm infection and positive samples underwent whole genome sequencing. Phylogenetic analysis was performed on 127 samples (25 DENV-1, 19 DENV-2, 70 DENV-3, and 13 DENV-4). We observed a serotype shift in 2014 and 2022. We detected the following genotypes per serotype for the wild-type (WT) DENV sequences: genotype IV (DENV-1), cosmopolitan (DENV-2), genotype I (DENV-3) and genotype IIa (DENV-4). WT DENV belonged to different genotypes versus the QDENGA strains and except for DENV-4, belonged to different genotypes versus the Dengvaxia strains. Comparing Dengvaxia vaccine sequences with WT DENV, we observed 23, 24, 34, and 9 positions with amino acid changes in the entire envelope protein, with 1, 5, 1, and 2 positions with amino acid changes identified among the important human monoclonal antibodies (mAbs) targeted epitope positions. We detected 24, 25, 36 and 12 positions with amino acid changes in the E protein with 0, 5, 1, and 2 positions with amino acid changes among the important mAbs targeted epitope positions for DENV-1, DENV-2, DENV-3, and DENV-4, respectively when comparing QDENGA vaccine sequences with the WT DENV. We showed low genotype complexity, genetically distinct clades and local evolution for DENV circulating in the Philippines.

## Author summary

Dengue virus has four distinct serotypes and each serotype is further divided into distinct genotypes. There are currently two licensed dengue vaccines, Dengvaxia and QDENGA and we compared the genotypes of the circulating wild type (WT) dengue viruses (DENV) versus the genotypes of the strains of the two dengue vaccines. We also analyzed whether there were any amino acid changes in selected locations which have been previously identified as targets by potent virus neutralizing human monoclonal antibodies (mAbs) which are considered important in vaccine development. We collected blood from patients diagnosed with dengue from 2014 to 2019 from the Philippines and sequenced the dengue RT-PCR positive samples. WT DENV all belonged to different genotypes versus the QDENGA vaccine and except for DENV-4, belonged to different genotypes versus the Dengvaxia strains. We detected several amino acid changes in the dengue envelope protein and in particular, among important mAbs targeted epitope positions among the 4 serotypes when we compared QDENGA and Dengvaxia vaccine sequences with WT DENV sequences. We observed that the DENV genotypes circulating in the Philippines from 2014–2019 had low complexity and the circulating clades were genetically distinct.

## Introduction

Dengue virus (DENV) of the *Flaviviridae* family has four distinct serotypes (DENV-1,-2,-3,-4) [1] and the extensive genetic diversity within each of the four serotypes contribute to its complexity as a disease. Before 1970, less than 10 countries had experienced severe dengue epidemics. However, in recent years, dengue has spread globally to more than a hundred tropical and subtropical countries [2,3] with hundreds of millions of DENV infectious estimated every year and with South-East Asia and South Asia consistently having the highest burden of disease from 1990–2019 [3–5]. The Philippines experienced its first dengue hemorrhagic fever (DHF) outbreak in 1953–1954 and this was followed by outbreaks in Bangkok, Thailand in 1958 and then by Malaysia, Singapore and Vietnam in the 1960s [6].

In May 2019, US Food and Drug Administration approved Dengvaxia (Sanofi Pasteur, France) for use among ages nine through sixteen and who have had previous laboratory-confirmed dengue infection and living in endemic areas [7]. However, approval was not extended for the vaccine’s use in travelers visiting a dengue endemic area [8]. QDENGA (Takeda, Tokyo) has been approved by the European Commission, United Kingdom and several countries in South America and Southeast Asia for use in individuals 6 to 45 years of age regardless of previous dengue exposure [9,10].

Dengvaxia efficacy among the four DENV serotypes was not well balanced with a combined study efficacy endpoint of 59.2% (52.3–65.0), combined efficacy against hospitalized dengue of 72.7% (62.3–80.3) and a combined efficacy against severe dengue of 79.1% (60.0–89.0) but had variable efficacy by serotype, serostatus and age [11–14]. Furthermore, there was a high relative risk of severe dengue among vaccinated seronegative individuals [14,15]. More recently, Takeda’s tetravalent dengue vaccine, QDENGA (TAK-003) or DENVax, completed a Phase 3 pivotal clinical trial which showed overall vaccine efficacy of 80.2% and 61.2% at 12 month and 54-month follow-up, respectively, with no evidence of disease enhancement among vaccine recipients [16,17]. Similar to Dengvaxia, serotype-specific VE of QDENGA among baseline seronegatives significantly varied with 3-year efficacy data showing no efficacy observed against DENV-3 and inconclusive results for DENV-4 due to the low number of DENV-4 infections detected [9,18,19]. Year by year analysis also showed that serotype dominance partially contributed to differences in VE against virologically confirmed dengue with efficacy observed to decline over time [19,20].

Both Dengvaxia and QDENGA are live attenuated tetravalent chimeric vaccines using recombinant DNA technology. Dengvaxia has a yellow fever 17D vaccine strain backbone with structural precursor membrane and envelope genes from each of the four DENV serotypes. The Dengvaxia parent strains were derived from the following isolates, DENV-1: Thailand PUO-359/TVP-1140 isolated in 1980, DENV-2: Thailand PUO-218 isolated in 1980, DENV-3: Thailand PaH881/88 isolated in 1988, and DENV-4: Indonesia 1228 (TVP-980) isolated in 1978 [14,21,22]. QDENGA is based on the DENV-2 PDK-53 attenuated virus backbone with DENV-DENV chimeras. The QDENGA parent strains were derived from the following isolates, DENV-1: Thailand 16007/PDK-13 isolated in 1964 [23], DENV-2: Thailand 16681/ PDK-53 isolated in 1964 [24], DENV-3: Philippines 16562 TC-7/ PGMK30 isolated in 1964 [25], and DENV-4: Indonesia 1036, isolated in 1976 [26,27].

Beginning of 2016, the Philippine Department of Health (DOH) launched a dengue mass vaccination campaign. Dengvaxia was administered to 880,464 nine to fourteen year-old children [28] in the National Capital Region or Metro Manila, Central Luzon (Region 3), Calabarzon (Region 4A). In April 2016, the first dengue vaccine dose was given with the subsequent second and third doses administered on October 2016 and April 2017, respectively (DOH, Philippines). In July 2017, the program was extended to Cebu province with around 149,023 children receiving a single dose only since in December of the same year, the vaccination program was discontinued [29].

An important aspect of dengue phylogenetic studies has been the dissection of the extent and structure of genetic variation among major intra-serotype lineages (genotypes) within each serotype [30]. A close antigenic match between circulating wild-type (WT) DENVs and vaccine strains may result to a greater estimated VE against circulating WT DENVs. The VE of Dengvaxia vaccine strains was estimated to be greater when circulating WT DENVs had shorter amino acid sequence distances compared to the vaccine strains [31]. Interestingly, this correlation between VE and amino acid distance was limited to the CYD14 (Asia) clinical trial but was not observed in the CYD15 (Latin America) clinical trial. Furthermore, a genotype-level VE association was observed within WT DENV-4 with serotype-specific analysis showing a significant decrease in VE for this particular serotype as amino acid distance increased compared to the DENV-4 vaccine insert. Sub-analysis though showed this effect to be restricted among 2 to 8 year olds [31,32].

There are still critical and fundamental gaps in our understanding of the evolutionary dynamics of DENV. It is important to understand the role of DENV genetic variability and its potential role and impact in the effectiveness of the dengue vaccine. It is important to determine whether the vaccination campaign resulted to sieve effects or exerted selection pressure resulting to selection of certain DENV lineages. Here we present a molecular evolutionary analysis and WT genetic diversity data of all four DENV serotypes circulating in Metro Manila from 2014 to 2019 and comparison of the extent of dengue genetic variation among important vaccine target epitopes between WT DENV strains and DENV vaccine strains.

## Materials and methods

### Ethics statement

The Armed Forces of the Philippines Health Service Command (AFPHSC) Research Ethics Committee approved the conduct of this study with registration number WRAIR#2464. All samples were consented for future research use and samples from patients 18 years old and above had written informed consent; parents or legal guardians provided written informed consent for children less than 18 years of age. Samples were coded with no associated personally identifiable information.

### Study Site and Sample collection

The study was conducted at V Luna General Hospital (VLGH), Armed Forces of the Philippines Medical Center (AFPMC), a tertiary care, military hospital located in Quezon City, Philippines. DENV reverse transcription polymerase chain reaction (RT-PCR) testing was performed at the Armed Forces of the Philippines Medical Center–Armed Forces Research Institute of Medical Sciences (AFPMC–AFRIMS) Collaborative Molecular Laboratory, VLGH, AFPMC. Whole genome sequencing (WGS) and bioinformatics analysis were done at the Department of Virology, Walter Reed Army Institute of Research–Armed Forces Research Institute of Medical Sciences (WRAIR-AFRIMS), Bangkok, Thailand. Patients ≥ 6 months old with a history of fever or measured temperature ≥ 38°C within 5 days of illness onset and clinically diagnosed with dengue by the attending physician based on the 2009 World Health Organization case classification were enrolled. Majority of the patients were from Quezon City, Metro Manila and neighboring Regions 3 (Central Luzon), and Region 4-A (CALABARZON) with few patients coming from Regions 1 (Ilocos), 5 (Bicol), 9 (Zamboanga Peninsula), 1 (Cordillera Administrative Region), and 10 (Northern Mindanao) (S9 Fig).

VLGH personnel used a service sample case report form to gather coded demographic and clinical data together with blood samples from patients presenting at the outpatient department or the emergency room. We randomly selected serum samples, representing each of the four DENV serotypes circulating per year from WT DENV RT-PCR positive samples collected from 2014 to 2019. The clinical sera samples used for sequencing were collected from laboratory confirmed dengue patients in the general area where dengue mass vaccinations occurred but who were not vaccinated with either Dengvaxia or QDENGA.

### RNA extraction and Nested RT-PCR

Blood samples were processed into serum aliquots and tested by DENV nested RT-PCR at the AFPMC–AFRIMS Collaborative Molecular Laboratory located within VLGH. Viral RNA was extracted from 140 μl of sera using QIAamp viral RNA mini kit (QIAGEN, Germany) following the manufacturer’s instructions. We used a semi-nested RT-PCR method modified from Lanciotti et al. as previously described [33,34] to determine the dengue serotype. For the semi-nested RT-PCR, the target of amplification is the capsid/PrM gene region. The first round RT-PCR products, amplified by forward primer D1 and reverse primer D2, are 511 base pairs (bp) in length. The second round PCR (nested) products, amplified by forward primer D1 and reverse primers TS1, TS2, TS3, and TS4 (specific to DENV-1 to 4, respectively), are 482 bp, 119 bp, 290 bp, and 392 bp in length, respectively. No PCR products were used for sequencing in this study and the diagnosis of dengue was based solely on nested RT-PCR testing of the acute specimens. Serum aliquots of DENV RT-PCR positive specimens were stored at −70°C and shipped on dry ice to AFRIMS in Bangkok, Thailand, for sequencing.

### DENV isolation and Whole Genome Sequencing (WGS)

Sera positive for DENV by nested RT-PCR were inoculated into freshly prepared monolayers of C6/36 cells grown in Minimum Essential Medium (MEM, GIBCO) and which contained 10% heat inactivated fetal bovine serum (HIFBS), 1% Glutamine and 1% Penicillin and streptomycin. Cells were cultured and underwent three passages until cytopathic effect was observed. Virus isolates, which were successfully amplified via cell culture, were subsequently used for WGS. For DENV WGS, viral RNA was extracted from 140 μl of cell culture virus isolate using QIAamp Viral RNA Mini Kit (QIAGEN, Valencia, U.S.A.) following manufacturer’s instructions. Extracted RNA was used in DENV-1 to 4 genome amplification following the method previously described by Baronti et. al., 2015 [35] in which four to five overlapping PCR fragments that covered DENV entire genome were amplified using DENV specific primers and SuperScript III One-Step RT-PCR System with Platinum *Taq* High Fidelity DNA Polymerase (Life Technologies Corporation, California, USA). PCR fragments were analyzed using a QIAxcel Advanced Systems (QIAGEN, Germany) and then purified by Agencourt AMPure XP beads (Beckman Coulter, USA). The purified-PCR products were quantified with a Qubit 3.0 Fluorometer using a Qubit dsDNA HS Assay Kit (Life Technologies, USA) followed by DNA library preparation. DNA libraries were prepared using a (QIAGEN, Germany) according to the manufacture’s instruction. The quality and quantity of DNA libraries were validated by a QIAxcel Advance Systems and a Qubit 3.0 Fluorometer, respectively. Pooled DNA libraries with optimized concentration were loaded into a MiSeq Reagent Cartridge 2x250 cycles (Illumina, USA) and sequenced on a MiSeq platform according to the manufacturer’s instruction (Illumina, USA). The paired-end reads were quality filtered and trimmed using BBTools v38. 22 [36] to remove adapter sequences and low quality bases/reads using the following criteria; trim quality = 30, minimum length = 100, and minimum average quality = 20. DENV whole genome sequences were generated from mapping trimmed reads against to the reference genome (LC128301.1 for DENV-1, KU509275.1 for DENV-2, KU509279.1 for DENV-3, and KC333651.1 for DENV-4) using BWA v0.7.17-r1188 [37]. Primer trimming and consensus sequences calling were performed using iVar v1.3.1 [38]. The consensus sequences were supported with a depth of coverage of ≥ 10 reads and base quality of ≥ 30.

### Phylogenetic analysis

A total of 204 published complete coding sequences (CDS) included 46, 59, 49, and 50 sequences of DENV-1 to 4, respectively and 338 envelope gene (E) sequences included 47, 61, 93, and 137 sequences of DENV-1 to 4, respectively, along with known and unknown genotypes collected from different countries and various years (1944 to 2019) were downloaded from GenBank. These GenBank sequences were then combined and aligned with sequences obtained from this study, and sequences or synthetic constructs derived from Dengvaxia and QDENGA as well as other dengue vaccine candidates including TDENV (WRAIR/GSK); TetraVax-DV (US NIH/ NIAID); and V180 (Merck) by using MAFFT v7.475 [39]. Maximum-likelihood trees of the complete CDS from all serotypes were constructed using IQ-Tree v2.1.2 [40] with 1,000 ultrafast bootstrap replicates and substitution model GTR+F+I+G4 for DENV-1, DENV-3, DENV-4 and TIM2+F+I+G4 for DENV-2. Maximum-likelihood trees of E gene sequences from all serotypes were constructed using IQ-Tree v2.1.2 with 1,000 ultrafast bootstrap replicates and substitution model TIM2+F+G4 for DENV-1, TN+F+I+G4 for DENV-2, and TIM2+F+I+G4 for DENV-3 and DENV-4. Trees were visualized using FigTree v1.4.2 (http://tree.bio.ed.ac.uk/software/figtree/). Percentage nucleotide and amino acid similarity among the WT DENV E gene sequences from our study were compared to both Dengvaxia and QDENGA using MEGA v.6.0 (www.megasoftware.net) [41].

*Sequence analysis and comparison of human mAb epitopes between vaccine strains and WT DENVs*.

We quantified the E-protein sequence differences between sequences of 5 dengue vaccine strains and WT DENV sequences from our study. We also performed sequence conservation analysis and described positions on the E protein where differences of amino acid residues between the WT DENV sequences from this study and that of the vaccine strains were found. This included analysis of selected epitope locations previously illustrated and identified as targets by potent virus neutralizing human monoclonal antibodies (mAbs) and considered important in immune correlate assays and vaccine development [32,42–50].

## Results

**Fig 1:**
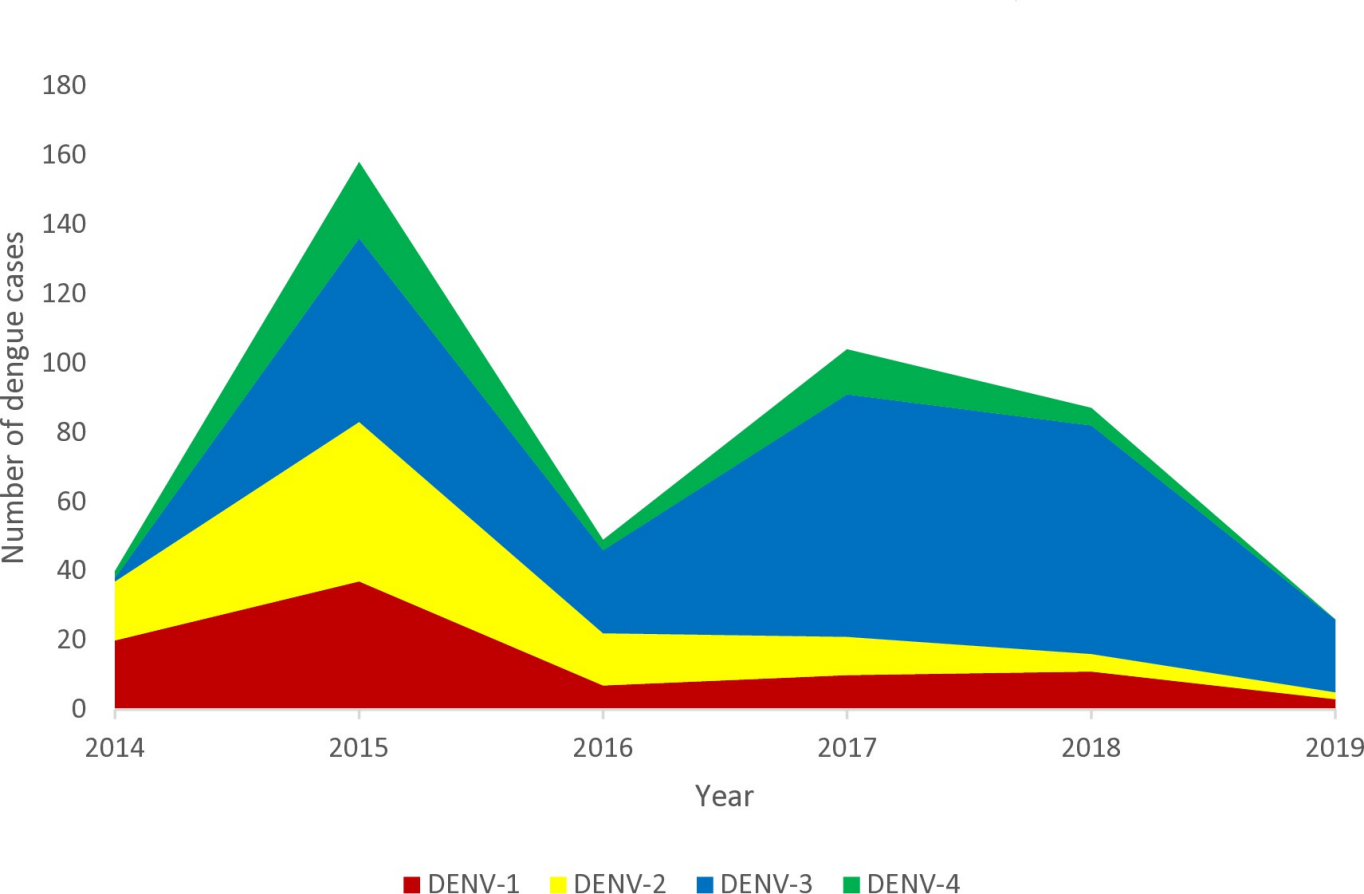
Dengue serotype distribution per year (N = 464).

One hundred and fifty serum samples, representing each of the four DENV serotypes circulating per year from 2014 to 2019 were selected from 464 WT DENV RT-PCR positive samples collected during the same period (Fig 1). One hundred twenty-seven (25 DENV-1, 19 DENV-2, 70 DENV-3, 13 DENV-4) out of the 150 serum samples were successfully amplified using cell culture for three passages and were subsequently used for WGS. DENV-3 was the dominant serotype from 2015 through 2019 (Fig 1). Except for 2017, DENV-4 showed the lowest prevalence from 2014 to 2019. We observed a serotype shift in 2014 wherein DENV-1 was the predominant serotype that year but was eventually replaced with DENV-2 and DENV-3 as the dominant serotypes in 2015. In 2019, we did not detect the DENV-4 serotype from our samples but this particular serotype was still circulating based on the Philippine national dengue surveillance (DOH, 2019).

Phylogenetic analyses of the E-gene and whole genome of the WT DENV sequences showed the following genotypes for each serotype: genotype IV (DENV-1), cosmopolitan (DENV-2), genotype I (DENV-3) and genotype IIa (DENV-4) (S1–S8 Figs). All WT DENV belonged to different genotypes versus the QDENGA strains and except for DENV-4, all WT DENV also belonged to different genotypes versus the Dengvaxia strains (Table 1 and S1– S8 Figs).

**Table 1:** Comparison of WT DENV genotypes (2014–2019) and vaccine strains based on E gene sequences.

DENV (GenBank Accession number)	Number of sequences	Genotype
DENV-1, WT	25	IV
DENV-1, Dengvaxia (KX239894)	1	I
DENV-1, QDENGA/DENVax (AF180818)	1	II
DENV-2, WT	19	Cosmopolitan
DENV-2, Dengvaxia (KX239895)	1	Asian I
DENV-2, QDENGA/DENVax (U87412)	1	Asian I
DENV-3, WT	70	I
DENV-3, Dengvaxia (KX239896)	1	II
DENV-3, QDENGA/DENVax (KU725665)	1	V
DENV-4, WT	13	IIa
DENV-4, Dengvaxia (KX239897)	1	IIa
DENV-4, QDENGA/DENVax (U18429)	1	IIb

WT DENV-1 circulating viruses belonged to the same genotype as the candidate vaccine strains for TDENV and TetraVax-DV (S1 and S5 Figs) but the WT DENV-2 belonged to a different genotype versus the TDENV and TetraVax-DV (S2 and S6 Figs). The WT DENV-3 circulating viruses belonged to the same genotype as TDENV but differed from TetraVax-DV genotype (S3 and S7 Figs). WT DENV-4 were classified under Genotype II, same as TDENV and TetraVax-DV, but differed from the genotype of the V180 vaccine strain (S4 and S8 Figs).

Within each serotype of WT DENV, DENV-1 had the lowest percentage amino acid similarity at 99.7% and DENV-4 had the highest percent amino acid identity at 99.9% (Table 2). The mean level percentage amino acid similarity of the genotype for each of the four serotypes of the WT DENV when compared to the Dengvaxia and QDENGA genotypes ranged from 97.3 to 99.2% and 96.6 to 98.4%, respectively (Table 2). In our study, the average amino acid similarity between the Dengvaxia and QDENGA vaccine strains compared to the E gene of the WT DENV circulating in the Philippines from 2014 to 2019 were 97.3%, (DENV-1) 97.4% (DENV-2), 98.0% (DENV-3), 99.2% (DENV-4) and 96.6% (DENV-1), 97.0% (DENV-2), 97.4% (DENV-3), and 98.4% (DENV-4), respectively (Table 2).

**Table 2:** Percentage similarity of nucleotide and amino acid identity of WT DENV E gene sequences (2014–2019) versus Dengvaxia and QDENGA.

Comparison	% Nucleotide Identity			% Amino Acid Identity		
	Min	Max	Average	Min	Max	Average
Within DENV-1 Genotype IV (25 WT sequences)	96.50	100.00	98.43	99.19	100.00	99.74
Between DENV-1 Genotype IV (WT) and Genotype I (Dengvaxia)	89.93	91.40	90.86	96.92	97.55	97.32
Between DENV-1 Genotype IV (WT) and Genotype II (QDENGA)	90.71	92.04	91.57	96.09	96.71	96.57
Within DENV-2 Cosmopolitan (19 WT sequences)	97.58	99.87	98.93	98.98	100.00	99.77
Between DENV-2 Cosmopolitan (WT) and Asian I (Dengvaxia)	91.68	92.78	92.19	96.71	97.55	97.44
Between DENV-2 Genotype IV (WT) and Asian I (QDENGA)	92.71	93.63	93.07	96.51	97.34	97.03
Within DENV-3 Genotype I (70 WT sequences)	97.06	99.52	98.48	99.39	100.00	99.83
Between DENV-3 Genotype I (WT) and Genotype II (Dengvaxia)	91.41	92.97	92.32	97.54	98.36	98.01
Between DENV-3 Genotype I (WT) and Genotype V (QDENGA)	92.20	93.81	93.20	96.91	97.74	97.39
Within DENV-4 Genotype II (13 WT sequences)	98.77	99.25	98.98	99.60	100.00	99.88
Between DENV-4 Genotype II (WT) and Genotype II (Dengvaxia)	95.41	95.85	95.69	98.78	99.39	99.22
Between DENV-4 Genotype II (WT) and Genotype II (QDENGA)	93.31	94.07	93.51	97.96	99.19	98.41

**Fig 2:**
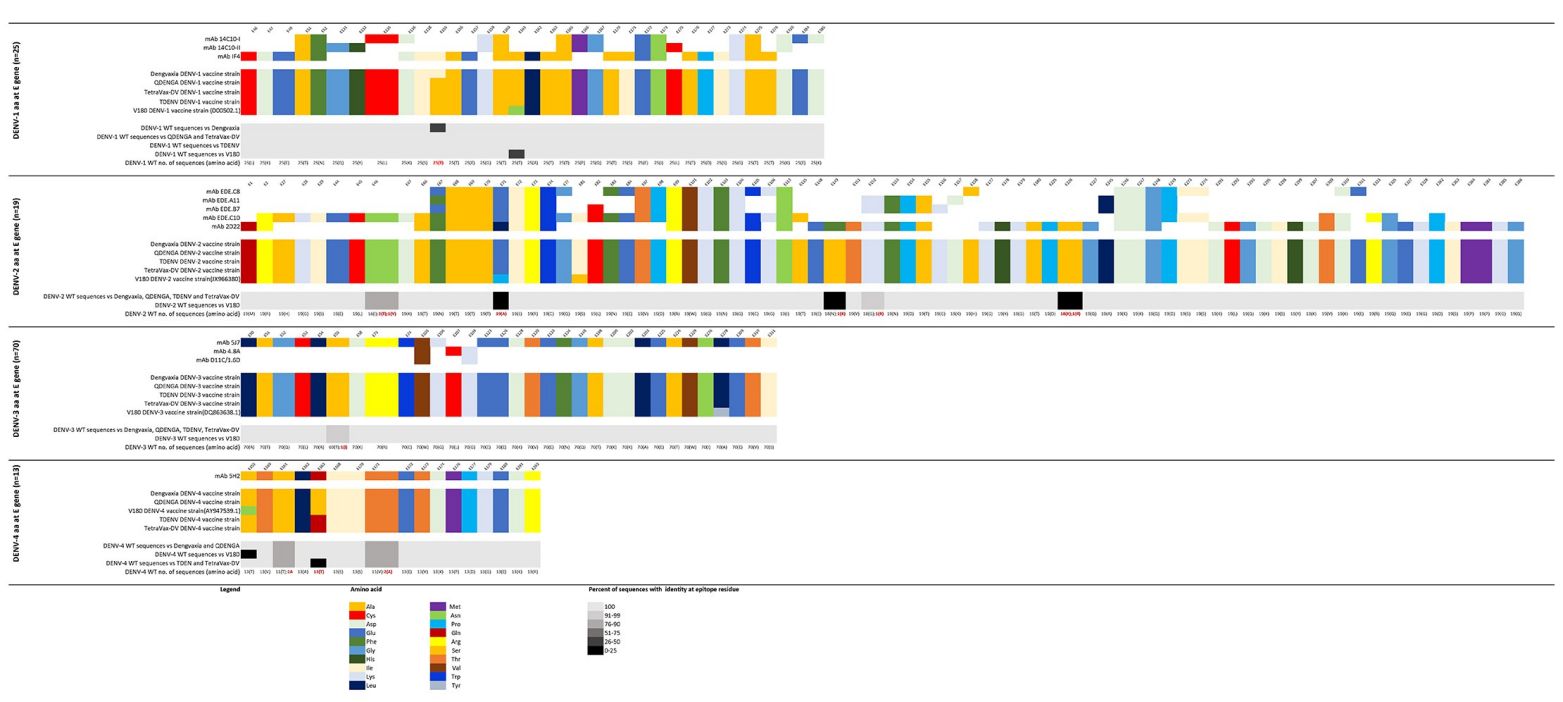
Sequence conservation between dengue vaccine components versus wild‐type dengue viruses at epitope locations targeted by virus neutralising human mAbs.

When we compared QDENGA vaccine sequences with the WT DENV sequences from this study, we detected 24, 25, 36 and 12 positions (S1– S4 Tables) with amino acid changes in the E protein with 0, 5, 1, and 2 positions with amino acid changes among the important mAbs targeted epitope positions for DENV-1, DENV-2, DENV-3, and DENV-4, respectively (Fig 2). Comparing Dengvaxia vaccine sequences with the WT DENV sequences from our study, we observed 23, 24, 34, and 9 positions (S5–S8 Tables) with amino acid changes in the entire envelope (E) protein, with 1, 5, 1, and 2 positions with amino acid changes identified among the important mAbs targeted epitope positions reported by Rabaa et.al. (2017) [32] for DENV-1, DENV-2, DENV-3, and DENV-4, respectively (Fig 2). When we compared the DENV WT DENV strains with the Dengvaxia vaccine sequences, the E protein had a total of 24 amino acid changes and DENV-2 had the most number of amino acid changes (n = 5) among the known mAbs targeted epitopes compared to the other DENV serotypes. When we compared our DENV WT sequences with the different dengue vaccine sequences using the mAbs targeted epitope positions (Fig 2), we observed for DENV-1, amino acid differences at E155 and E161 for Dengvaxia and V180, respectively. Among all the 4 DENV serotypes, our DENV-2 WT sequences had the most number of amino acid changes detected in the mAbs targeted epitope positions with E71, E149 and E226 different for all dengue vaccine sequences. Position E81 was different for V180 only while 3/19 and 1/19 DENV-2 WT sequences were different at positions E46 and E152 (Fig 2). For DENV-3, only 1 out of the 70 WT sequences had a different amino acid at position E55. For DENV-4, the amino acid for all 13 WT DENV-4 strains were different at E155 for V180 and at E163 for both TDEN and TetraVax while 2/13 DENV-4 WT sequences differed at positions E161 and E171 for all the dengue vaccines. The amino acid positions, which differed between Dengvaxia, QDENGA and other dengue vaccine candidate strains, as compared to the WT DENV, were dispersed across the E gene and we did observe clustering at any particular structural domain (S1–S8 Tables).

## Discussion

The Philippines continues to be hyperendemic for dengue, with all four DENV serotypes circulating from 2014 to 2018. Intra-serotypic antigenic differences have been observed with neutralizing monoclonal and polyclonal antibodies in-vitro and these differences were postulated to be related to vaccine development [51–53]. The complex interplay between the population dynamics of serotype-specific immunity and viral genetics have been known to be a major driver in determining disease severity and outcome [53]. A genotype mismatch between the genotype used in a dengue vaccine and the circulating genotypes of a DENV serotype theoretically may have an impact on the efficacy of a vaccine though breakthrough infections from the CYD14/15 trials showed absence of a direct relationship between VE and genetic similarity between Dengvaxia strains and wild-type strains [32]. The E protein is commonly used for phylogenetic analyses because it is located on the exterior of the virus, making it a key target for host immune responses and antigenic variation. Its involvement in the virus’s evolution provides valuable insights into genetic diversity and transmission patterns. Additionally, the E protein is widely used for genotyping, and there is an extensive amount of sequence data available in the GenBank database, facilitating comprehensive comparative analyses. In our study, there was high intra-serotypic E protein similarity among all our WT DENV, with percent amino acid similarity ranging from 99.3 to 99.9 across the four DENV serotypes. We did not detect any shift in the predominant genotype during the duration of our study (2014 to 2019), with only one major genotype detected for each DENV serotype. Based on literature search, circulating DENV-1 and DENV-3 genotypes in the Philippines are unchanged since 1974 and 1997, respectively. Genotype I was the predominant lineage for DENV-4 prior to 2014 and we detected a genotypic shift to genotype II around 2014. For DENV-2, the Cosmopolitan lineage has been the predominant genotype since 2005. Circulating DENV genotypes tend to remain relatively stable as shown by similar predominant genotypes over the course of our study and when compared to Genbank Philippine DENV sequences from previous years. Based on the DENV-1 (S1 and S5 Figs) and DENV-3 (S3 and S7 Figs) phylogenetic trees, we observed that two different clades of DENV-1 genotype IV and DENV-3 genotype I co-circulated during 2015 to 2019 though we did not observe clade extinction and replacement events across all DENV serotypes in the same period.

A close antigenic match between circulating WT DENV and vaccine strains may result to a higher estimated VE against circulating WT DENV. Rabaa et al. (2017) [32] reported that genotype-level VE association against DENV-4 was statistically significant with VE against DENV-4 genotype I found to be significantly lower versus genotype II in all ages, but sub-group analysis showed similar VE between genotype I and II among 9–16 y.o. In a serotype-specific analysis done by Juraska et al. 2018 [31], they reported that VE decreased with the degree of amino acid dissimilarity between the vaccine strains and disease causing DENVs. They observed this especially in DENV-4, but after accounting for differential VE by serotype, this effect seemed to be limited to younger children (2 to 8 y.o.) who had lower baseline seropositivity and possibly less broadly protective immune response [31]. In our study, while the E protein of WT DENV-3 and DENV-4 were most similar to that of Dengvaxia and QDENGA vaccine strains, E protein of WT DENV-1 and DENV-2 had the highest average percentages of amino acid difference compared to both vaccine strains.

Amino acid differences among the circulating WT DENV and the strains from the Dengvaxia and QDENGA vaccine may have an impact on vaccine effectiveness, particularly if the changes are in epitopes targeted by DENV neutralizing mAbs [32]. When we compared Dengvaxia and other dengue vaccine candidate sequences with our WT DENV sequences, particularly, at the important mAbs targeted epitope positions indicated by Rabaa et. al., 2017 [32], we detected few amino acid changes in these positions across all four DENV serotypes (Fig 2). When we compared the E protein of the WT DENV strains with the QDENGA vaccine sequences, DENV-3 had the highest number of amino changes (n = 37), though for this particular serotype, we only detected 1 amino acid change among the known mAbs targeted epitopes. In contrast, when we compared the DENV-2 WT DENV strains with the QDENGA vaccine sequences among the known mAbs targeted epitopes, we detected the highest number of amino acid changes (n = 5) but the number of amino acid changes in the E protein was lower (n = 25) versus DENV-3. The relatively higher number of amino acid changes in the E protein of DENV-3 may be related to the lack of DENV-3 VE observed in QDENGA among seronegatives but despite the number of amino acid changes in the E-protein (n = 25) and in the mAbs targeted epitopes (n = 5), high VE was still observed in the DENV-2 serotype specific analysis [19]. This is most probably because of the DENV-2 PDK-53 backbone which may have elicited a higher serotype-specific immunity reaction compared to the other DENV serotypes.

Based on the generated E-gene and whole genome phylogenetic trees (S1–S8 Figs) of our sequences, genotypes of WT DENV 1–3 which circulated in the Philippines from 2014 to 2019 were all different from both the Dengvaxia and QDENGA vaccine strains. Both Dengvaxia and QDENGA DENV-4 vaccine strains belonged to genotype II, same as the DENV-4 WT strains. The Dengvaxia DENV-4 vaccine strains more closely matched the circulating DENV WT strains which were IIa versus the QDENGA vaccine strains which were IIb.

Sequencing of genomes directly from clinical serum samples is ideal but due to low viral loads, we had to perform analyses on sequences from viruses amplified in C6/36 cells for 3 passages. Fung et al (2021) [54], observed that intra-host single nucleotide variants (iSNVs) with frequencies ≥ 5% were often maintained during passage, sample diversity did not significantly differ between the clinical sample and isolates, and were representative of their direct sample parental viruses [54] though this study was limited to DENV-1 and DENV-2 serotypes. Mutations we detected may have been introduced in viruses passaged through C6/36 cells and though low-passage number has minimal effects to the dengue virus consensus sequence [55], an increase in the number of isolate passages can result to an increase in the number of mutations in the consensus sequences [56] and may have resulted in removal of low-frequency variants [54].

Though a reliable correlate of protection against dengue is still lacking [57], extra-neutralizing antibody functions may play an important protective role against symptomatic disease [57,58]. Furthermore, dengue vaccine efficacy trials have indicated that neutralizing antibodies do not always translate in clinical efficacy [11] and further investigations are needed in the functions of extra-neutralizing antibodies. Though previous studies showed evidence correlating VE against specific DENV genotypes and amino acid mismatches between the vaccine component and specific genotypes [44], it is still equivocal whether these differences will actually translate to any clinical significance. A major caveat is that what we currently know about the amino acid differences between vaccine strains and WT DENV at mAbs targeted B cell epitopes that we describe here, is still incomplete and some are still hypothetical. Thus we cannot directly infer that there will be a resultant change in antigenicity or immunogenicity due to the differences since functional assays of antibody binding are still needed. Finally, the WT DENV sequences in our study were also closely related to publicly available contemporary DENV sequences from the Philippines, indicating ongoing local evolution.

### Limitations

This paper has certain limitations. The sequences from this study were collected in just a span of six consecutive years which may not be enough to detect significant changes given the average mutation rate of DENV estimated at 7.5 × 10^− 4^ mutations/position/year [59]. The collection did not extensively cover different geographic parts of the Philippines and hence, we might not have detected rare genotypes. We also cannot directly assess whether these specific mutations will have an impact on VE since the specimens did not come from individuals given Dengvaxia or QDENGA, and as such, we cannot perform virus genotype-specific estimates of VE or correlate potential immune escape mechanisms due to differences in epitope sites or amino acid residue changes. All of the sequencing data were obtained from dengue viruses which underwent three passages in C6/36 cells and it is possible that some of the mutations detected may have been selected by the cell passages.

### Recommendations

Amino acid changes, especially in certain epitopes targeted by virus neutralizing mAbs, may affect the VE of Dengvaxia or any other future dengue vaccines. We recommend sequencing directly from samples if the viral loads are sufficiently high since there is a possibility of mutations being introduced during cell passages. We also recommend that functional antibody binding assays be conducted among E gene epitope positions where we have observed specific mutations since some of these may be targets of virus neutralizing mAbs. We also recommended continued surveillance to monitor WT DENV evolution, diversity, local genotype variation and mapping the epitopes targeted by virus neutralizing mAbs among contemporary DENV serotypes and genotypes and using this data in the evaluation and development of licensed dengue vaccines and dengue vaccine candidates, respectively.

## Supporting information

S1 Table 25 WT DENV-1 E-genes (1485 bp, 495 amino acid) compared to QDENGA (TAK-003) (AF180818). (XLSX)

S2 Table 19 DENV-2 WT E-genes (1485 bp, 495 amino acid) compared to QDENGA (TAK-003) (U87412). (XLSX)

S3 Table : 70 DENV-3 WT E-genes (1479 bp, 493 amino acid) compared to QDENGA (TAK-003) (KU725665). (XLSX)

S4 Table 13 DENV-4 WT E-genes (1485 bp, 495 amino acid) compared to QDENGA (TAK-003) (U18429). (XLSX)

S5 Table 25 WT DENV-1 E-genes (1485 bp, 495 amino acid) compared to Dengvaxia (KX239894). (XLSX)

S6 Table 19 DENV-2 WT E-genes (1485 bp, 495 amino acid) compared to Dengvaxia (KX239895). (XLSX)

S7 Table 70 DENV-3 WT E-genes (1479 bp, 493 amino acid) compared to Dengvaxia (KX239896). (XLSX)

S8 Table 13 DENV-4 WT E-genes (1485 bp, 495 amino acid) compared to Dengvaxia (KX239897). (XLSX)

S1 Fig Phylogenetic tree of 130 DENV-1 E gene sequences. Maximum-likelihood tree of 130 E gene sequences (1485 nt) included 25 sequences from the Philippines study (red), and 105 sequences from GenBank (4 vaccine strains in green, 4 from Philippines in blue, and 97 from other countries in black). The tree was constructed using IQ-Tree with substitution model TIM2+F+I+G4 and 1,000 ultrafast bootstrap replicates. (TIF)

S2 Fig Phylogenetic tree of 155 DENV-2 E gene sequences. Maximum-likelihood tree of 155 E gene sequences (1485 nt) included 19 sequences from the Philippines study (red), and 136 sequences from GenBank (4 vaccine strains in green, 20 from Philippines in blue, and 112 from other countries in black). The tree was constructed using IQ-Tree with substitution model TIM2+F+I+G4 and 1,000 ultrafast bootstrap replicates.
(TIF)

S3 Fig Phylogenetic tree of 192 DENV-3 E gene sequences S4 Fig: Phylogenetic tree of 172 DENV-4 E gene sequences. Maximum-likelihood tree of 192 E gene sequences (1479 nt) included 70 sequences from the Philippines study (red), and 122 sequences from GenBank (4 vaccine strains in green, 56 from Philippines in blue, and 62 from other countries in black).The tree was constructed using IQ-Tree with substitution model TIM2+F+I+G4 and 1,000 ultrafast bootstrap replicates.
(TIF)

S4 Fig Phylogenetic tree of 172 DENV-4 E gene sequences.Maximum-likelihood tree of 172 E gene sequences (1485 nt) included 13 sequences from the Philippines study (red), and 159 sequences from GenBank (4 vaccine strains in green, 67 from Philippines in blue, and 88 from other countries in black). The tree was constructed using IQ-Tree with substitution model TIM2+F+I+G4 and 1,000 ultrafast bootstrap replicates.
(TIF)

S5 Fig Phylogenetic tree of 71 DENV-1 coding sequences. Maximum-likelihood tree of 71 CDS sequences (10179 nt) included 25 sequences from the Philippines study (red), and 46 sequences from GenBank (3 vaccine strains in blue, 2 from Philippine in green, and 41 from other countries in black). The tree was constructed using IQ-Tree with substitution model GTR+F+I+G4 and 1,000 ultrafast bootstrap replicates.
(TIF)

S6 Fig Phylogenetic tree of 78 DENV-2 coding sequences. Maximum-likelihood tree of 78 CDS sequences (10176 nt) included 19 sequences from the Philippines study (red), and 59 sequences from GenBank (2 vaccine strains in blue, 18 from Philippine in green, and 39 from other countries in black). The tree was constructed using IQ-Tree with substitution model TIM2+F+I+G4 and 1,000 ultrafast bootstrap replicates.
(TIF)

S7 Fig Phylogenetic tree of 119 DENV-3 coding sequences. Maximum-likelihood tree of 119 CDS sequences (10173 nt) included 70 sequences from the Philippines study (red), and 49 sequences from GenBank (2 vaccine strains in blue, 5 from Philippine in green, and 42 from other countries in black). The tree was constructed using IQ-Tree with substitution model GTR+F+I+G4 and 1,000 ultrafast bootstrap replicates.
(TIF)

S8 Fig Phylogenetic tree of 63 DENV-4 coding sequences. Maximum-likelihood tree of 63 CDS sequences (10167 nt) included 13 sequences from the Philippines study (red), and 50 sequences from GenBank (3 vaccine strains in blue, 4 from Philippine in green, and 43 from other countries in black). The tree was constructed using IQ-Tree with substitution model GTR+F+I+G4 and 1,000 ultrafast bootstrap replicates.
(TIF)

S9 Fig Location of dengue cases according to numbers and location. Map created with Datawrapper (https://app.datawrapper.de/) using supplementary data csv files RegionPhilippines and MetroManilaCities. The maps use OpenStreetMap https://www.openstreetmap.org/copyright which is licensed under the Creative Commons Attribution-ShareAlike 2.0 license (CC BY-SA 2.0).
(TIF)

S1 Data Metro Manila Cities (data used to create S9 Fig map showing location of dengue cases according to cities in Metro Manila). (XLSX)

S2 Data Region Philippines (data used to create S9 Fig map showing location of dengue cases according to regions in the Philippines). (XLSX)
