# Supplementary figures and images for: Genetic diversity of dengue virus circulating in the Philippines (2014–2019) and comparison with dengue vaccine strains
Source: PLoS Negl Trop Dis. 2024 Dec 19;18(12):e0012697. doi: 10.1371/journal.pntd.0012697 (PMC11698566; doi:10.1371/journal.pntd.0012697)

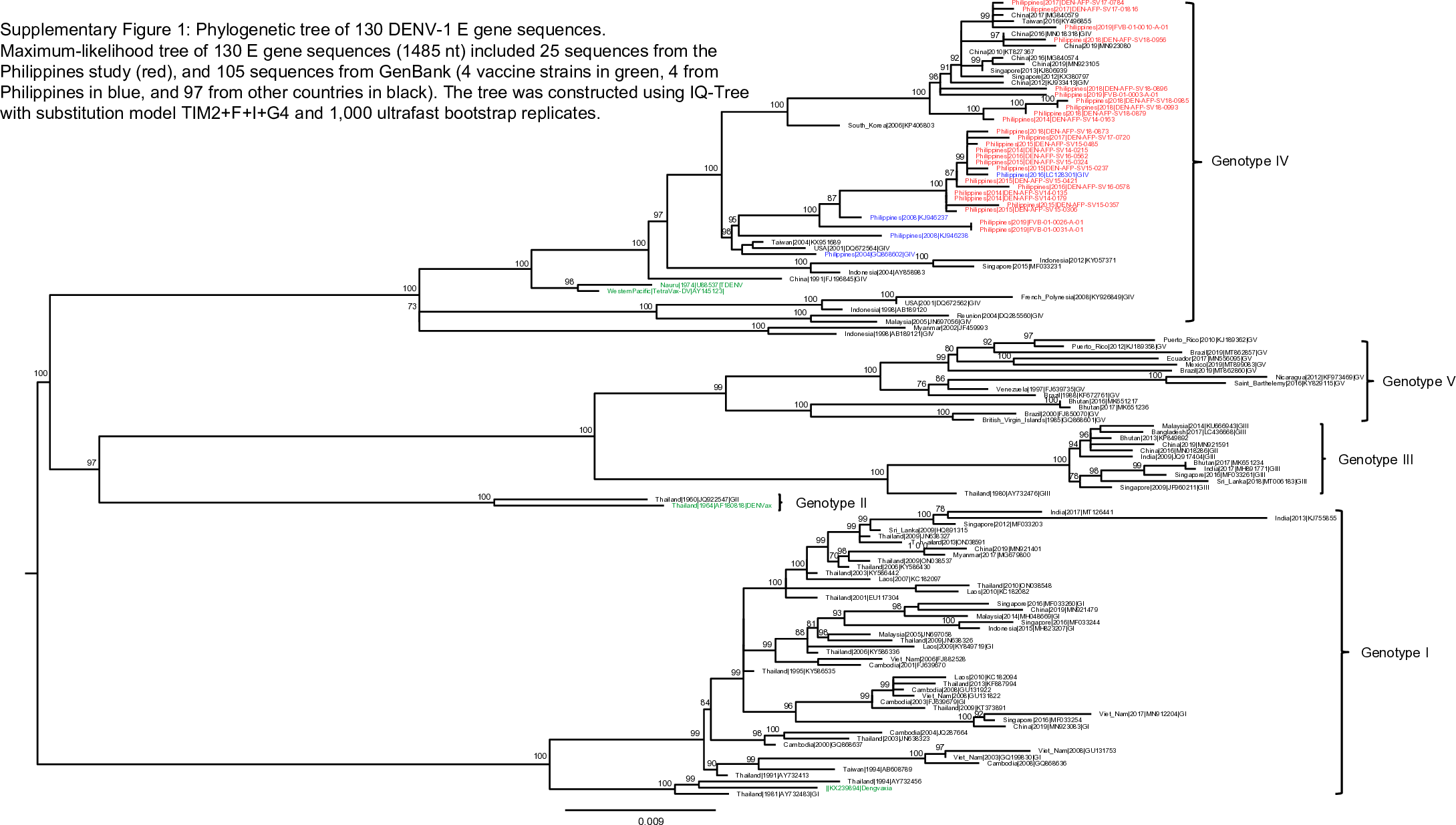

Supplement: S1 Fig — (TIF) [file pntd.0012697.s009.tif]

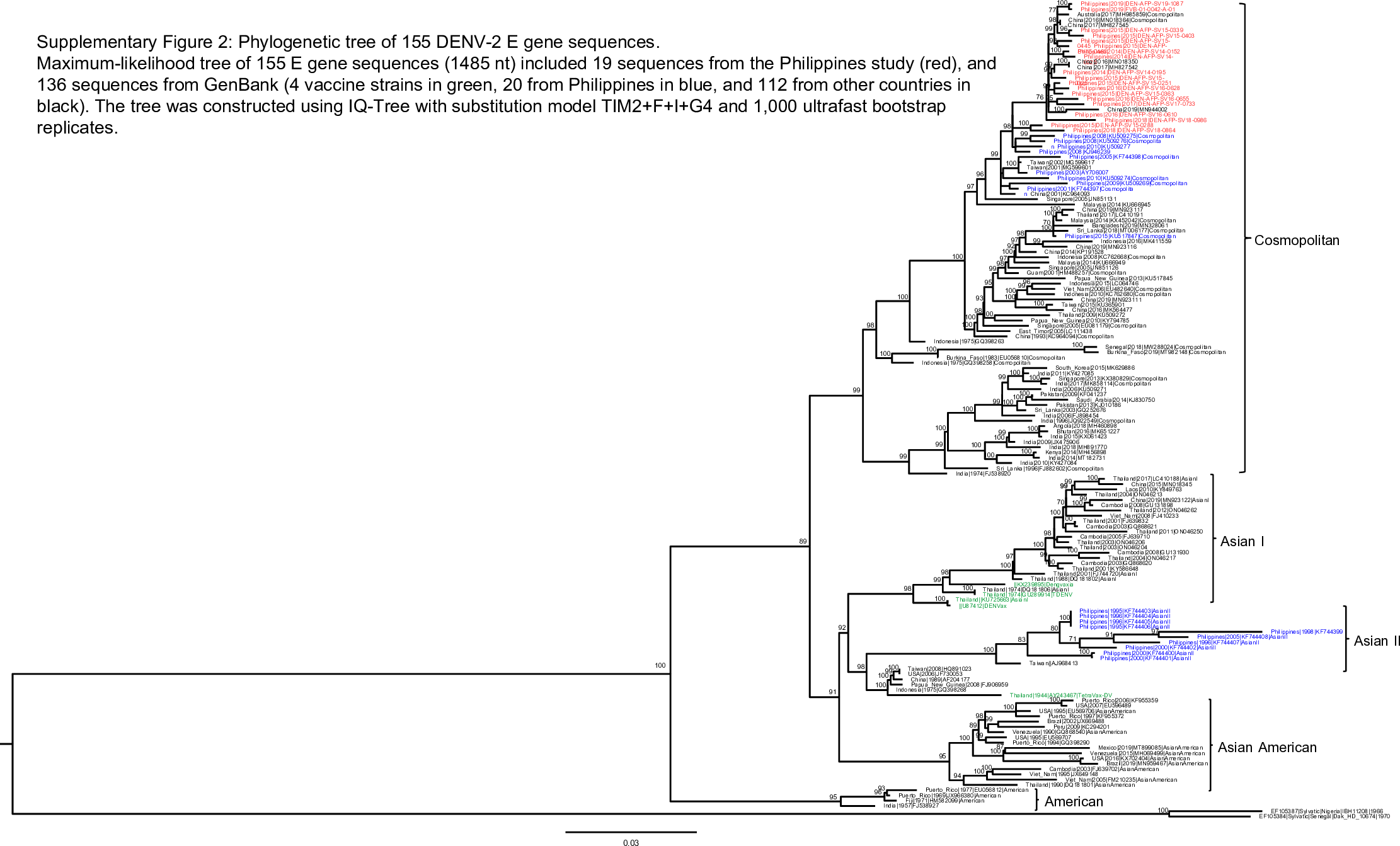

Supplement: S2 Fig — Maximum-likelihood tree of 155 E gene sequences (1485 nt) included 19 sequences from the Philippines study (red), and 136 sequences from GenBank (4 vaccine strains in green, 20 from Philippines in blue, and 112 from other countries in black). The tree was constructed using IQ-Tree with substitution model TIM2+F+I+G4 and 1,000 ultrafast bootstrap replicates. (TIF) [file pntd.0012697.s010.tif]

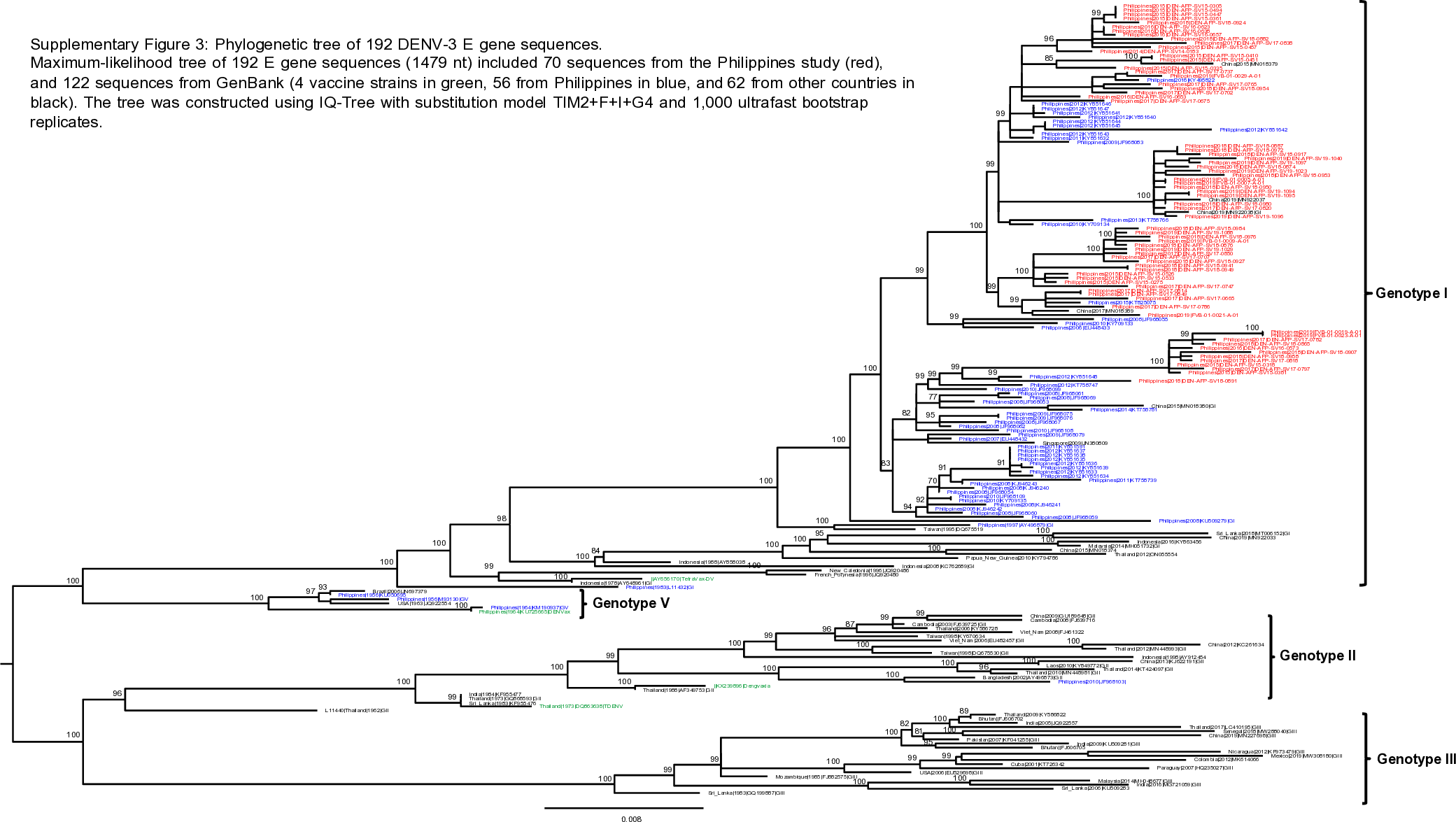

Supplement: S3 Fig — Maximum-likelihood tree of 192 E gene sequences (1479 nt) included 70 sequences from the Philippines study (red), and 122 sequences from GenBank (4 vaccine strains in green, 56 from Philippines in blue, and 62 from other countries in black).The tree was constructed using IQ-Tree with substitution model TIM2+F+I+G4 and 1,000 ultrafast bootstrap replicates. (TIF) [file pntd.0012697.s011.tif]

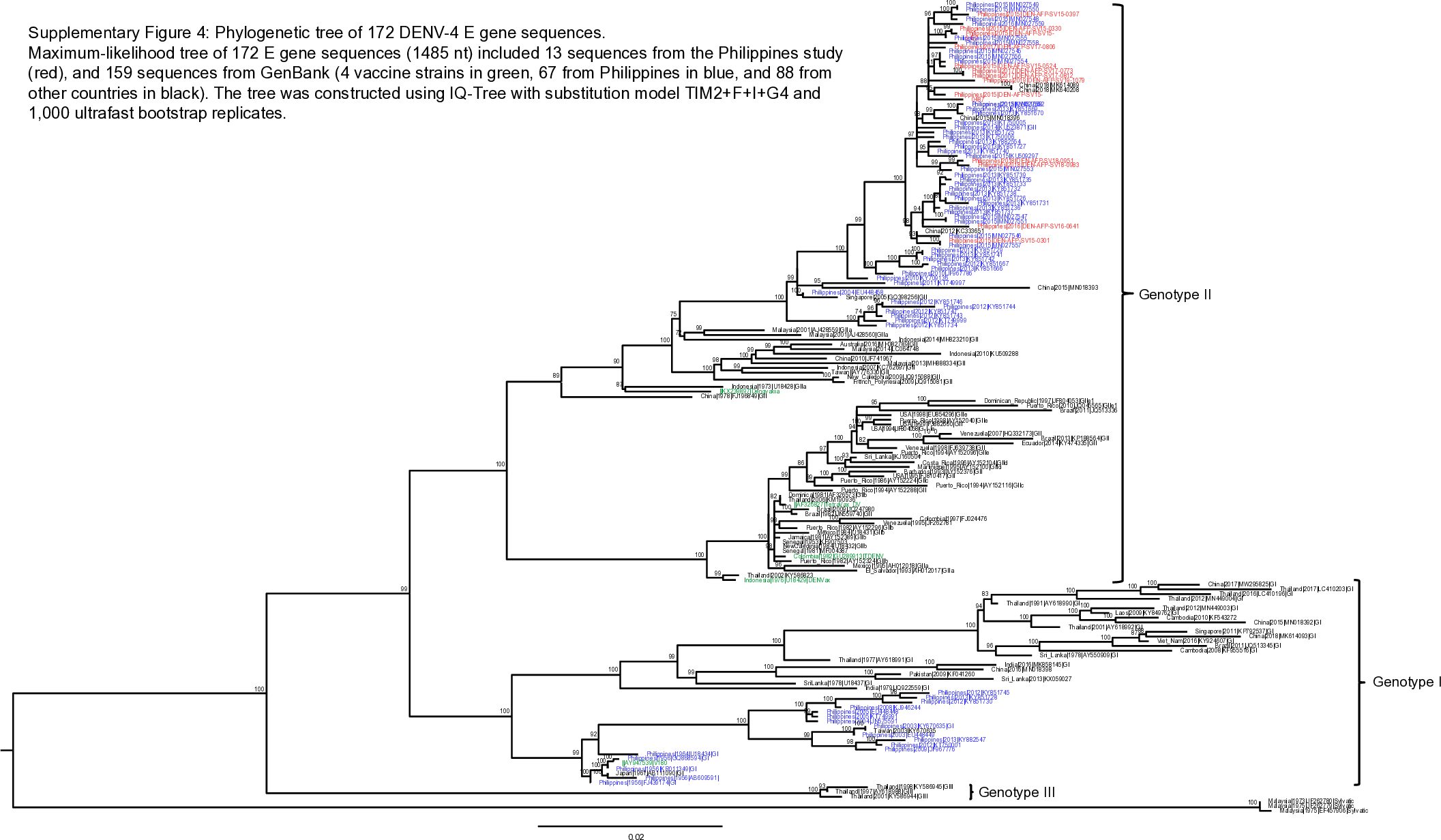

Supplement: S4 Fig — The tree was constructed using IQ-Tree with substitution model TIM2+F+I+G4 and 1,000 ultrafast bootstrap replicates. (TIF) [file pntd.0012697.s012.tif]

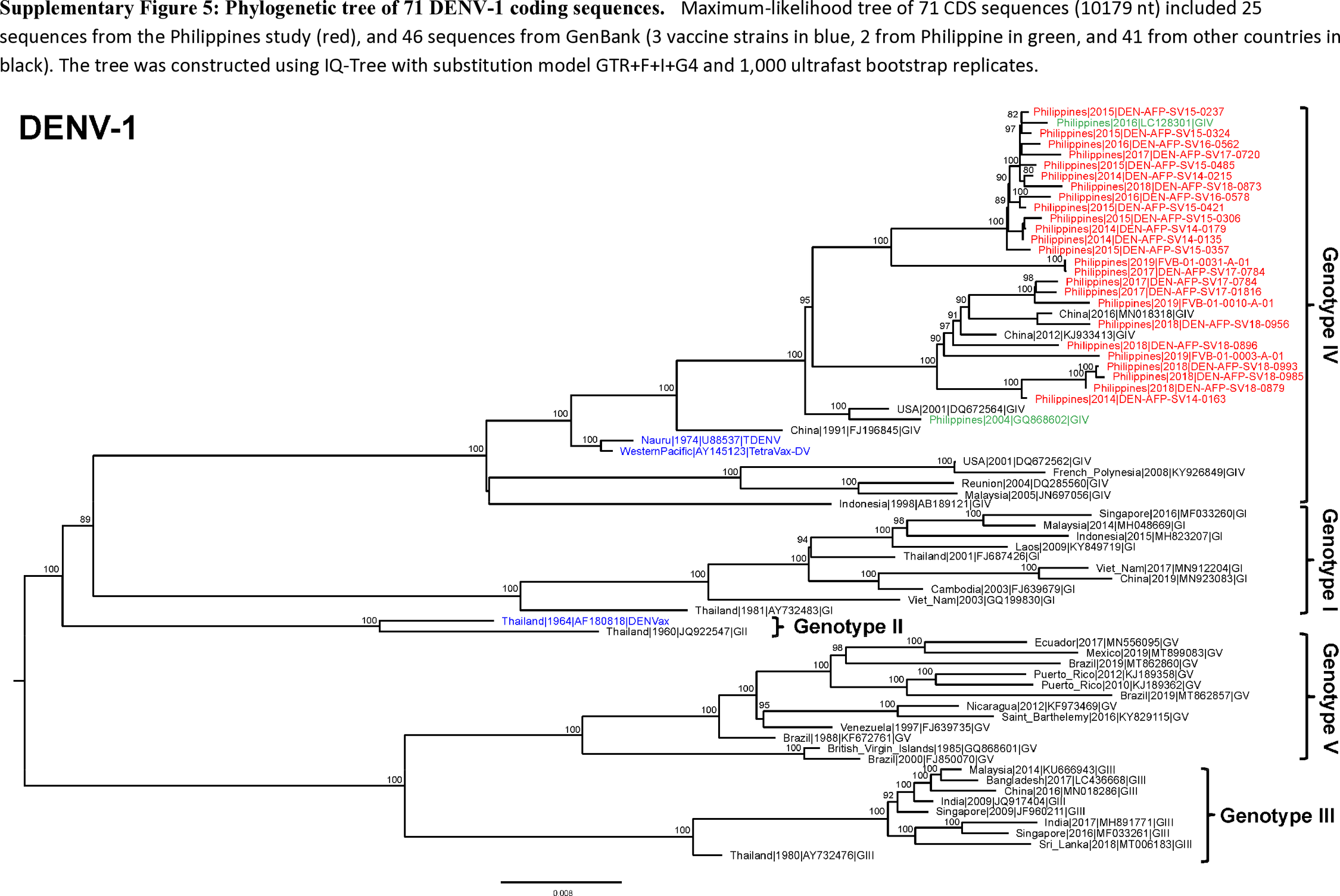

Supplement: S5 Fig — Maximum-likelihood tree of 71 CDS sequences (10179 nt) included 25 sequences from the Philippines study (red), and 46 sequences from GenBank (3 vaccine strains in blue, 2 from Philippine in green, and 41 from other countries in black). The tree was constructed using IQ-Tree with substitution model GTR+F+I+G4 and 1,000 ultrafast bootstrap replicates. (TIF) [file pntd.0012697.s013.tif]

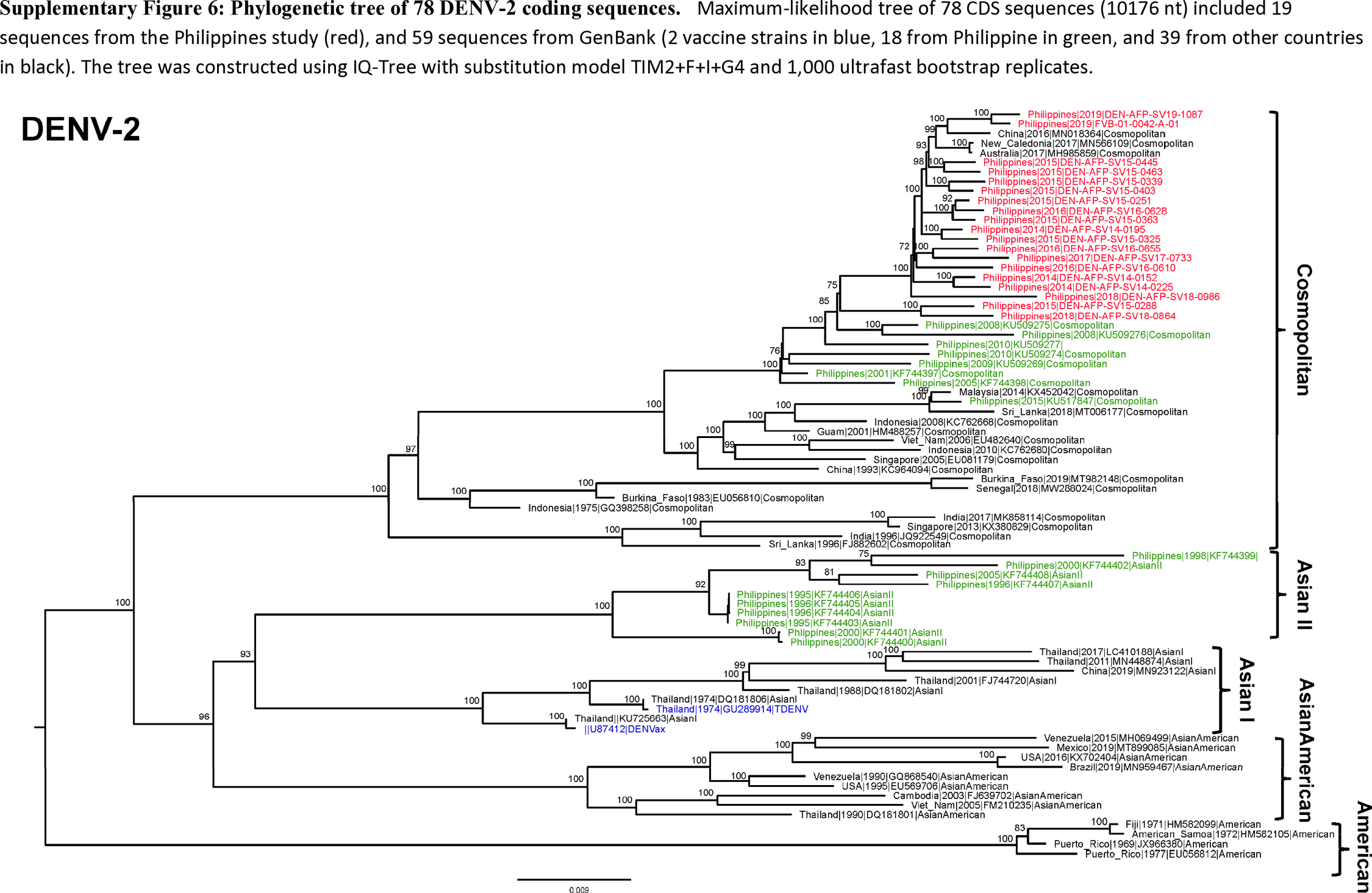

Supplement: S6 Fig — Maximum-likelihood tree of 78 CDS sequences (10176 nt) included 19 sequences from the Philippines study (red), and 59 sequences from GenBank (2 vaccine strains in blue, 18 from Philippine in green, and 39 from other countries in black). The tree was constructed using IQ-Tree with substitution model TIM2+F+I+G4 and 1,000 ultrafast bootstrap replicates. (TIF) [file pntd.0012697.s014.tif]

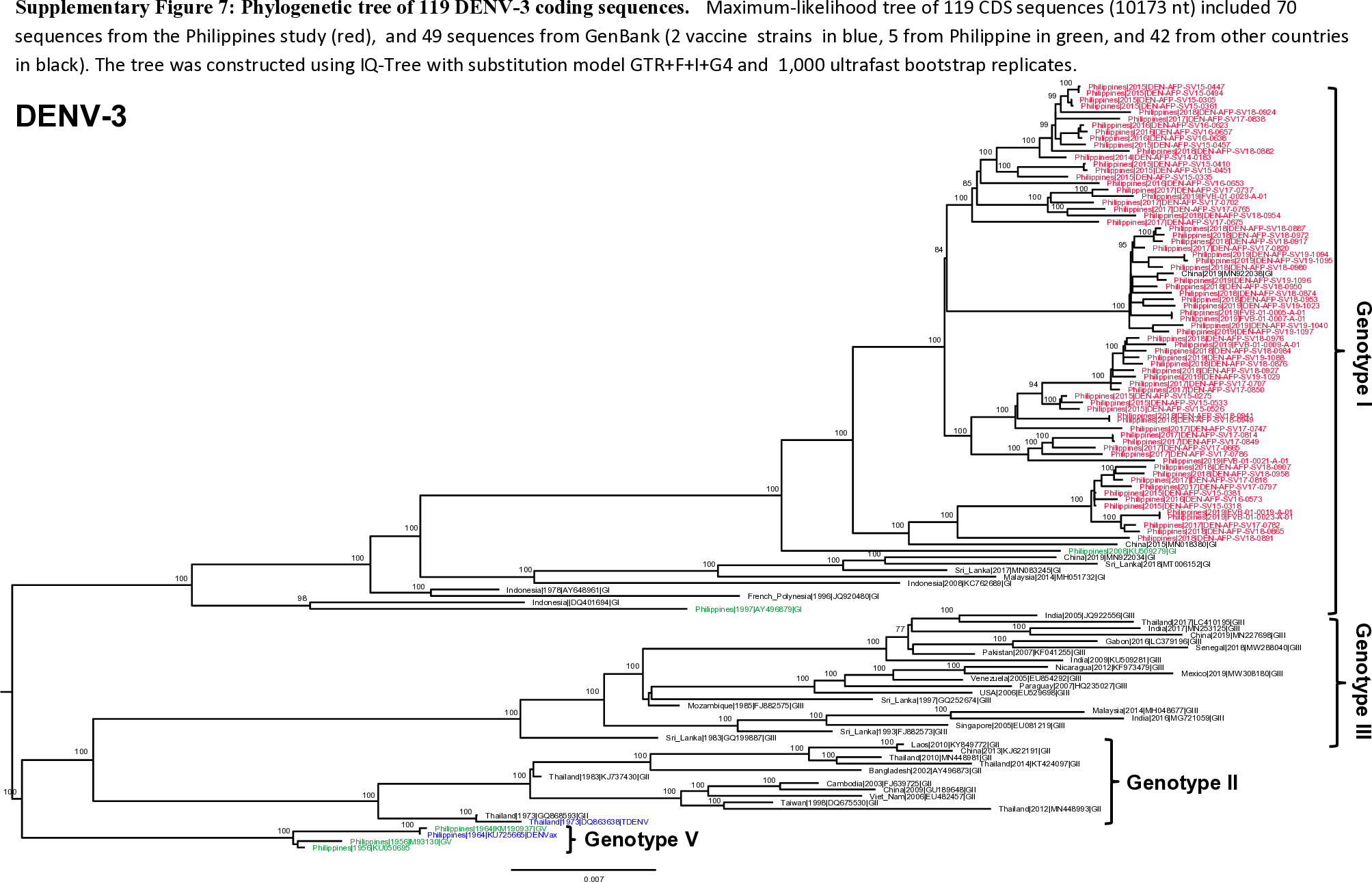

Supplement: S7 Fig — Maximum-likelihood tree of 119 CDS sequences (10173 nt) included 70 sequences from the Philippines study (red), and 49 sequences from GenBank (2 vaccine strains in blue, 5 from Philippine in green, and 42 from other countries in black). The tree was constructed using IQ-Tree with substitution model GTR+F+I+G4 and 1,000 ultrafast bootstrap replicates. (TIF) [file pntd.0012697.s015.tif]

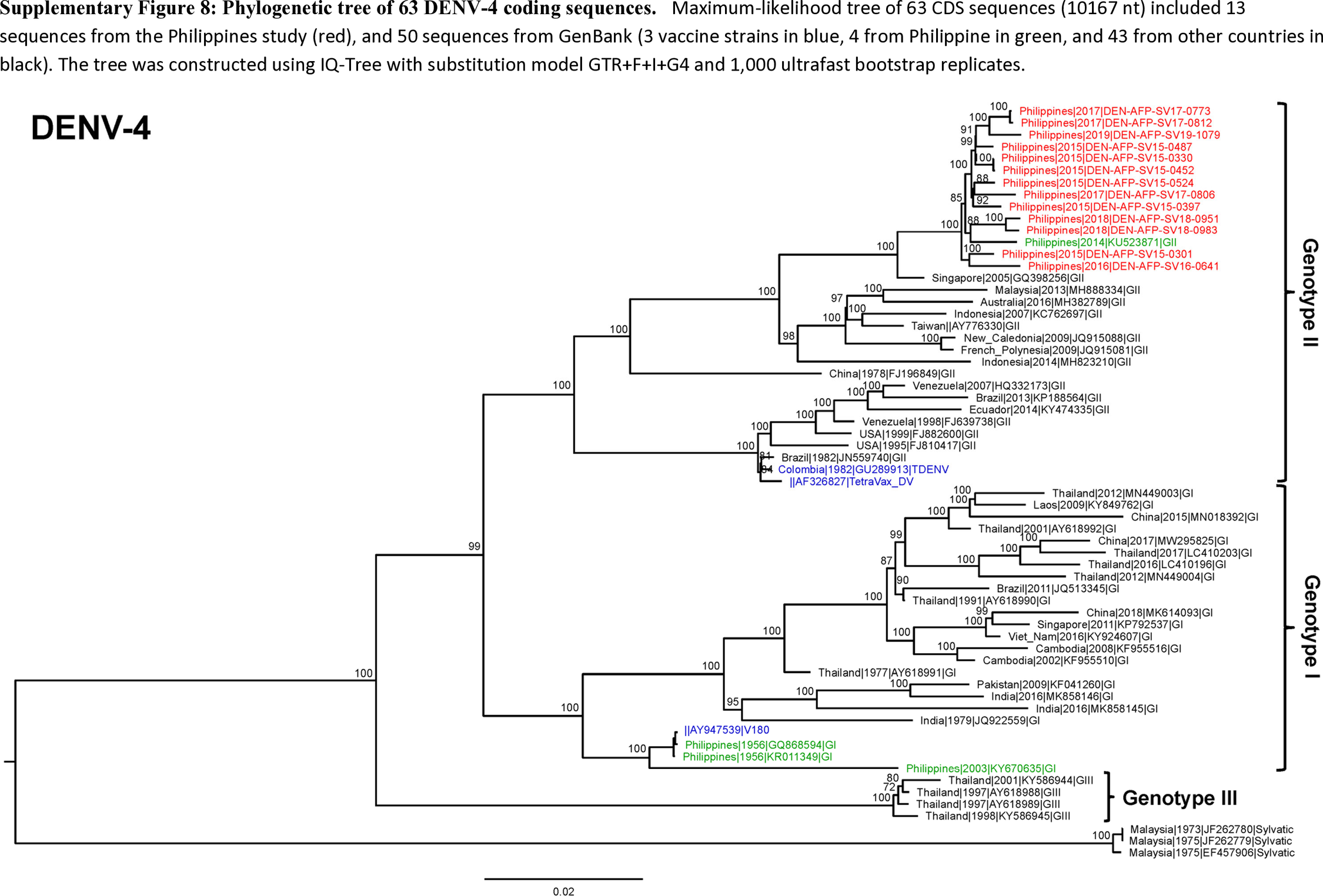

Supplement: S8 Fig — Maximum-likelihood tree of 63 CDS sequences (10167 nt) included 13 sequences from the Philippines study (red), and 50 sequences from GenBank (3 vaccine strains in blue, 4 from Philippine in green, and 43 from other countries in black). The tree was constructed using IQ-Tree with substitution model GTR+F+I+G4 and 1,000 ultrafast bootstrap replicates. (TIF) [file pntd.0012697.s016.tif]

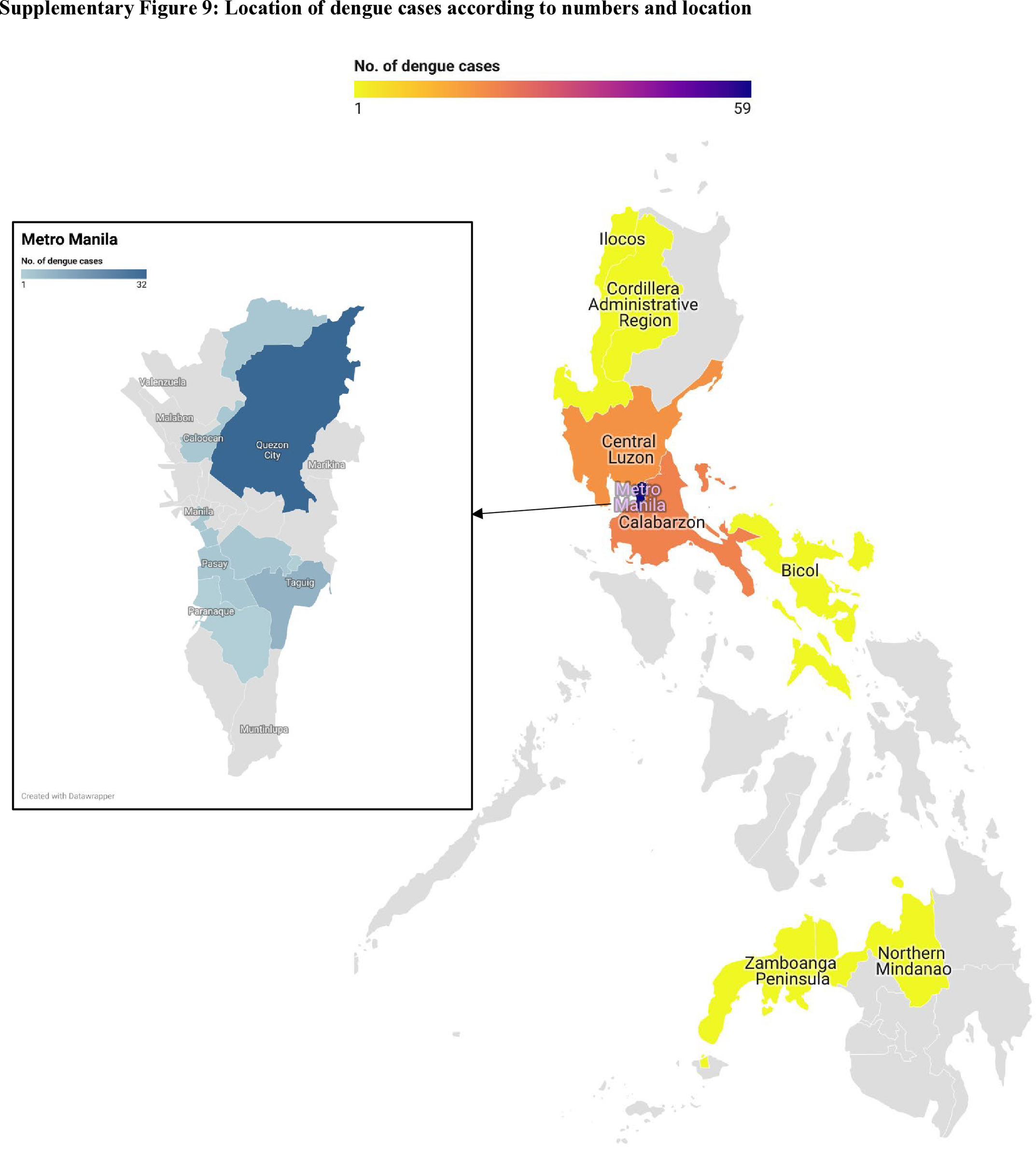

Supplement: S9 Fig — Map created with Datawrapper (https://app.datawrapper.de/) using supplementary data csv files RegionPhilippines and MetroManilaCities. The maps use OpenStreetMap https://www.openstreetmap.org/copyright which is licensed under the Creative Commons Attribution-ShareAlike 2.0 license (CC BY-SA 2.0). (TIF) [file pntd.0012697.s017.tif]
